# Effect of Plant Population and Inorganic Fertilizer on Growth, Phenology, Lodging, and Yield of Maize

**DOI:** 10.1155/sci5/6695323

**Published:** 2025-06-16

**Authors:** Terefe Tagesse, Addisalem Mebratu, Worku Mengesha

**Affiliations:** ^1^Plant Science Department, College of Agriculture and Natural Resource, Wolkite University, Wolkite, Ethiopia; ^2^Horticulture Department, College of Agriculture and Natural Resource, Wolkite University, Wolkite, Ethiopia

**Keywords:** blended fertilizer, economic benefit, environment, intrarow spacing, yield

## Abstract

Maize is the most important food security crop in Ethiopia. This study assessed the impact of inorganic blended NPSB (19% N, 38% P_2_O_5_, 7% S and 0.1% B) fertilizer rates and intrarow spacing (IRS) on growth, phenology, lodging characteristics, yield, and yield components of maize. Field trials were carried out at two locations in Kembata Tembaro Zone, southern Ethiopia during 2021 main cropping season. A factorial combination of three IRS (20, 25, and 30 cm) and five blended NPSB fertilizer rates (0, 50, 100, 150, and 200 kg·ha^−1^) was used in the experiment using a randomized complete block design replicated three times. At both sites, 150 kg·ha^−1^ NPSB rate produced the highest yields, 6.8 and 7.6 t·ha^−1^, respectively. However, maize hybrid did not result in significant yield gain to NPSB application over 150 kg·ha^−1^ NPSB at either site. The longest days to anthesis (AD) (88 days) and days to silking (DS) (98.83 days) were observed from the control treatment at 20 and 30 cm IRS. The longest days to physiological maturity (DPM) were measured from 200 kg·ha^−1^ NPSB at both sites. At both sites, an increase in applied NPSB dose resulted in increased root lodging (RL) and stem lodging (SL) magnitude. Likewise, the highest plant population resulted in the highest RL and SL. The economic analysis found that using 150 kg NPSB·ha^−1^ along with 100 kg·ha^−1^ resulted in a financial benefit of 88,957.52 Birr·ha^−1^ with an MRR of 1364.34% in Mugunja, and 98,318 Birr ha^−1^ with an MRR of 2933.16% in Lalo site. Therefore, 150 kg NPSB ha^−1^ at 30 cm IRS is recommended for optimum yield of maize in the study areas.

## 1. Introduction

Maize (*Zea mays* L.) stands as Ethiopia's foremost and extensively cultivated crop, second only to Tef (*Eragrostis tef* Trotter) in terms of land area dedicated to its cultivation [1]. Because of how widely it is used in the agricultural industry, it is recognized as a crop with a high agricultural economic value [2]. In Ethiopia, maize is produced in more than 2.9 million hectares of land [1] with an average productivity of 4.0 tonnes per hectare and an overall production of about 10.2 million tonnes [3]. Despite being a significant cereal crop, maize has a national average yield of only 3.2 t·ha^−1^ which is far lower than the global average yield of 5.2 t·ha^−1^ [3, 4]. Improved varieties, optimal fertilizer application, and optimum plant spacing have the potential to unleash the productivity of maize [5].

Previous studies have documented the responses of maize hybrids to fertilizer applications [5, 6]. According to Belay and Adare [7], maize demonstrates a high yield potential and responds favorably to fertilizer treatments. The optimal application of fertilizers generally enhances maize grain production and related yield components. However, in Ethiopia, improved maize cultivars are often grown below recommended agronomic practices, which contribute to the subpar yields of these improved varieties [8].

Hybrid maize displays heightened fertilizer responsiveness compared to traditional varieties, with performance shaped by genetic diversity, agroecological factors, and soil nutrient dynamics [9, 10]. While optimized practices maximize grain and biomass yields through increased nutrient absorption utilization and increased seed production [11]. However, subpar nutrient management hinders productivity, necessitating precision strategies [6]. Blended fertilizers mitigate localized nutrient gaps, as shown by Chimdessa [12], and aligning hybrids with site-specific fertilization unlocks their genetic potential, showcasing the synergy of genetics and tailored agronomy for sustainable gains [6].

Plant spacing significantly influences maize growth, yield, and yield components [8, 9]. High density fosters competition, potentially lowering productivity, whereas reducing plant numbers per unit area underutilizes production inputs [7]. Thus, for a specific maize genotype cultivated within defined environmental and management conditions, increasing plant density up to the optimal level has been shown to boost yield [13].

The availability of resources and a hybrid's tolerance for intraspecific competition both affect optimal density [14]. In order to maximize the absorption of solar radiation, some modern maize hybrids are planted at higher plant densities than the previous cultivars since they can endure stressors better [15]. However, high plant density above the optimum causes stresses on the plants, while low plant density reduces grain output [11, 12]. Inter- and intrarow spacing (IRS) both affect plant density [16].

Farmers in southern Ethiopia, including those in the Hadero district, typically adhere to uniform planting spacing and fertilizer recommendations for maize varieties, regardless of the variations in how each maize cultivar responds to factors like planting density, nutrient needs, and environmental conditions. This standardized approach could potentially result in less-than-optimal performance of the improved maize varieties. Plant population and fertilizer application significantly influence maize hybrid performance by balancing competition and nutrient availability. In Ethiopia, high-density planting (76,923 plants ha^−1^) with 150 kg/ha NPSB fertilizer boosted yields to 9393 kg/ha in hybrids like BH-546 [17, 18]. Meta-analyses show that increasing both nitrogen rates and plant density can raise yields by up to 152% on fertile soils, though excess density without adequate fertilization reduces grain quality [19, 20]. Optimal setups, such as 66,666 plants ha^−1^ with 150 kg·ha^−1^ NPSB, achieved 9.7 t·ha^−1^ for maize hybrid BH-546 in Bako [17]. Tailored strategies for specific hybrids, BH-546 emphasize the need to match management with genotype, soil, and agroecology for better yield and profit [21].

Tailoring fertilizer application and plant spacing to local conditions is key for optimal and sustainable production [22]. Yet in the Hadero Tunto district, NPSB rates and plant populations rely on national blanket recommendations even though maize cultivars have distinct nutrient and density needs. Research indicates that optimizing these factors can boost yields by 15%–25% [18, 20]. For instance, high-performing cultivars BH-546 have achieved higher grain yield (GY) under hybrid-specific practices [17]. Yasabu [23] further showed that targeted, site-specific recommendations can significantly enhance maize hybrid yields over blanket practices. Therefore, this study examines the effects of varying planting densities and NPSB fertilizer rates on maize growth, lodging, yield, and yield components under rain fed conditions in Hadero Tunto district, Southern Ethiopia.

## 2. Materials and Methods

The field experimental studies and data collection on maize were conducted following standard procedures and in compliance with applicable institutional, national, and international guidelines.

### 2.1. Study Sites Description

**Figure 1:**
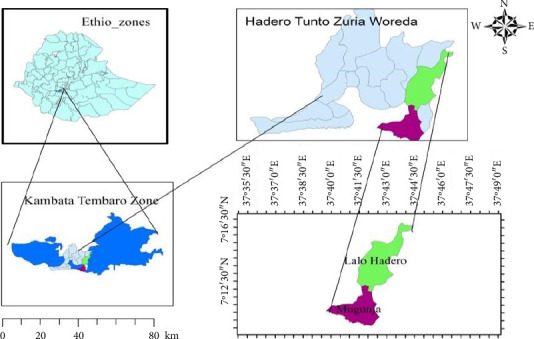
Map of the study sites.

Field experiments were conducted during the 2021 main cropping season at two maize-growing sites, Lalo (1710 masl; 1500 mm annual rainfall) and Mugunja (1650 masl; 1200 mm annual rainfall), in the Hadero Tunto Zuria District, Kembata Tembero Zone, Central Ethiopia Regional State (Figure 1).

### 2.2. Experimental Materials

The study utilized the widely cultivated hybrid maize variety *Shone* (PHB30G19), which was released in 2006 by Pioneer Hi-Breed Seeds Ethiopia and is popular in the district. Urea (46% N) was used as a nitrogen source, while blended NPSB fertilizer (18.9% N, 37.7% P_2_O_5_, 6.95% S, and 0.1% B) served as a source of nitrogen, phosphorus, sulfur, and boron for the study.

### 2.3. Treatments and Experimental Design

At each location, a 3 × 5 factorial treatment combination involving three IRS (20, 25, and 30 cm), resulting in three plant densities (66,666; 53,333; and 44,444 plants ha^−1^), and five rates of blended NPSB fertilizer (no fertilizer (control), 50, 100, 150, and 200 kg·ha^−1^). A randomized complete block design with each treatment replicated three times was used.

### 2.4. Trial Management

Each experimental plot consisted of six rows, each 4.5 × 3 m long; with gross plot area of 13.5 m^2^ and net plot area of 3 × 3 m. An IRS of 75 cm was kept for each plot. A distance of 0.5 and 1 m was kept between adjacent replications and plots, respectively. Two maize seeds per hole were sown on 21 April, 2021, and 23 April, 2021, at *Lalo* and *Mugunja* sites, respectively, and thinned at 2-3 leaf stage, giving the required population of 66,666, 53,333, and 44,444 plants·ha^−1^ for 20, 25, and 30 cm spacing, respectively. A basal fertilizer application of the full dose of blended NPSB fertilizer as per the treatments (except the control where no NPSB fertilizer was applied) was applied during planting. In all plots except for the control, the full recommended dose of urea (100 kg·ha^−1^) was applied in two separate applications: half during planting and the remaining half five weeks after sowing through side placement. For the control treatment, no fertilizer was administered. All other necessary agronomic practices were carried out uniformly across all experimental plots.

### 2.5. Data Collection and Measurements

Data collection was carried out in accordance with standard procedures for maize.

#### 2.5.1. Phenology

Days to silking (DS) was counted as the number of days between planting and the time at which 50% of the plants in a plot begin to emerge silk. Days to anthesis (AD) are determined by counting the days between planting and the day at which half of the plants in a plot have shed pollen. The difference between DS and AD was used to calculate anthesis silking interval (ASI). Number of days to physiological Maturity (DPM) was recorded as the number of days from planting to the formation of a black layer at the point of attachment of the kernel with the cob by 75% of the maize plants in the net plot area.

#### 2.5.2. Growth Characteristics

Plant height (PH) (cm) was measured as the distance from the base to the first tassel branch, while ear height (EH) (cm) was measured from the base to the upper ear node. Both measurements were taken from five randomly selected plants in the central rows of each plot 2 weeks after flowering (pollen shed).

### 2.6. Lodging Characteristics

Stalk lodging (SL) was determined based on the percentage of plants with broken stalks at or below the greatest ear node, following Badu-Apraku et al. [22] method. Root lodging (RL) was determined as the percentage of plants leaning more than 30° from the vertical upright position in the net plot area one week before harvest [22]. Data for both SL and RL were collected a week prior to harvest.

### 2.7. Ear Characteristics

Number of ears per plant (EPP) was computed by dividing the total ears harvested by the number of plants from the plot area, with each ear counted if it contained at least one fully developed grain. Ear width (EW) was measured in mm using a digital Vernier caliper on five randomly chosen ears, measured at the mid-way along the length. Ear length (EL) was determined as the ear length (cm) from base to tip, also averaging five ears.

### 2.8. Above Ground Biomass (AGBM), GY, and Harvest Index (HI)

Total AGBM (t ha^−1^) was determined from plants harvested from net plot area at both sites, after sun drying for 7 days to a constant weight and the result was converted to ton per hectare basis. GY (t ha^−1^) was determined on field weight basis from the net plot area and standardized at 12.5% moisture content, and 80% shelling percentage was assumed for estimating the GY and converted to t·ha^−1^ [22]. To determine grain moisture content (%), five representative cobs were selected and grains removed from their cobs. The moisture content of the grains was measured with digital moisture tester (Model PM-450, Kett Electric Laboratory).

(1) $$\begin{array}{c} \text{Grain yield }\left(t/\text{ha}\right)=\left[\frac{\left.\text{Fresh ear weight }\left(\text{kg}/\text{plot}\right)\times 10\times \left(100-\text{MC}\right)\times 0.8\right)}{\left(\left(100-\text{adjusted MC}\right)\times \text{Plot Area}\right.}\right], \end{array}$$

where MC is the actual moisture content of grain at harvesting, and adjusted MC in percentage (%), 0.8 is the shelling coefficient, and plot area refers to net plot area (middle four rows of each plot). HI (%): was determined as the ratio of GY to total AGBM (in kg) and multiplied by 100% at harvest from the net plot area each plot.

### 2.9. Soil Sampling and Analysis

Soil samples were collected once before planting using a zigzag pattern from 10 spots in the experimental field at a depth of 0–20 cm, employing an auger. A composite sample weighing 1 kg was prepared. Similarly, a 1 kg sample of farmyard manure was collected for chemical analysis. The collected soil and FYM samples were composited, air-dried at room temperature in the shade, and ground to pass through a 2 mm sieve. The composited sample was then placed in a polythene bag and submitted to the Wolkite regional soil laboratory for further analysis. Standard laboratory procedures were followed to determine soil texture, soil pH, cation exchange capacity (CEC), total nitrogen, exchangeable potassium, total organic carbon (OC), available phosphorus, and available sulfur.

The pH of the soils was determined by diluting the soil with water in a 1:2.5 ratio. Total nitrogen content was determined according to the method outlined by Tekalign [24]. CEC was determined by leaching the soil with 1 M ammonium acetate, washing with ethanol, measuring the adsorbed ammonium displaced by sodium [25]. Available phosphorus assessed using the Olsen method [26]. Available sulfur (meq/L SO_4_^−2^) was determined by the monocalcium phosphate extraction method [27], and available particle size (soil texture) was determined using the hydrometer method as described in Sertsu and Bekele [25].

### 2.10. Data Analysis

Analysis of variance (ANOVA), combined across locations was performed on plot means for individual traits with PROC GLM in SAS using a Random statement with TEST option of SAS version 9.3 [28]. For the combined analysis, the PROC MIXED procedure of SAS was used; where, NPSB, IRS, and their interactions were considered as fixed effects; while location and replication nested within location were considered as random effects. The significant differences among the treatments were detected by the least significant differences (LSD) at 0.05 probability level [29]. LSD was determined using the pooled error mean square. The statistical model used for the ANOVA across locations for this study is:

(2) $$\begin{array}{c} Y_{jjkl}=\mu +L_{i}+R_{j\left(i\right)}+{S_{k}+F}_{l}+{\left(S\times F\right)}_{kl}+{\left(L\times S\right)}_{ik}+{\left(L\times F\right)}_{il}+{\left(L\times S\times F\right)}_{ikl}+\varepsilon ijkl, \end{array}$$

where *Y*_*jjkl*_: is Observed response for the *k*th spacing, *l*th fertilizer rate, in the *j*th replication of the *i*th location; *μ*: is the overall mean; *S*_*k*_: is effect of the *k*th IRS; *F*_*l*_: effect of the *l*th NPSB fertilizer rate (kg·ha^−1^); (*S* × *F*)_*kl*_: interaction between IRS and fertilizer rate; *L*_*i*_: effect of the *i*th location; *R*_*j*(*i*)_: effect of the *j*th replication nested within the *i*th location; (*L* × *S*)_*ik*_, (*L* × *F*)_*il*_, (*L* × *S* × *F*)_*ikl*_: location interactions with IRS and fertilizer rates and *εijkl*: residual error.

### 2.11. Partial Budget Analysis

The economic viability of applying a blended NPSB fertilizer alongside different planting populations for maize production was examined in accordance with the procedures outlined in CIMMYT [30]. Variable costs, including seed, NPSB blended fertilizer, labor costs at the time of planting (May 2021), were taken into account. These costs comprised the prices of seed (40.00 ETB per kilogram), NPSB fertilizer (23.00 ETB per kilogram), and Urea (18.00 ETB per kilogram) as of March 2021. Additionally, the payment of labor costs for NPSB usage (3 persons per hectare, each at 75 ETB per day) was based on prevailing rates in the Hadero District. Other input costs and production practices, such as land preparation, planting, weeding, harvesting, transportation, and security, were assumed to be uniform across all treatments or plots. To adjust for typical farmer yields, the average yield was reduced by 10%, as outlined in CIMMYT [30]. The price of maize, as received by farmers upon sale after harvest, was determined based on the market price of maize near the study sites in Hadero. To pinpoint the treatments offering the highest returns on the farmers' investment, marginal analysis was conducted on nondominated treatments. For a treatment to be deemed a beneficial choice for farmers, the marginal rate of return (MRR) needed to fall within the range of at least 50%–100% [30]. Nevertheless, some researchers have advocated for a more conservative approach, recommending an MRR of 100% as realistic [31].

MRR (%) was calculated by dividing change in net benefit (ΔNB) by change in total variable cost (ΔTVC) as MRR (%) = (ΔNB/ΔTVC) × 100 as suggested by CIMMYT [30].

## 3. Results and Discussion

### 3.1. Soil Physical and Chemical Characteristics

Table 1 presents the findings from pre-sowing soil sample laboratory analysis of the experimental locations concerning specific physicochemical properties. Soil texture at the Mugunja site was identified as clay loam, while sandy loam characterized the Lalo site. According to Tisdale et al. [32], the soil pH at both sites fell within the moderately acidic range, conducive to maize cultivation. The analysis revealed low organic carbon levels at both locations, consistent with Landon's [33] classification. Additionally, available phosphorus was found to be low at both the Mugunja and Lalo sites [24].

### 3.2. Phonological and Growth Parameters

#### 3.2.1. Phenology

##### 3.2.1.1. AD, DS and ASI

Across environments, AD, DS, and the ASI were significantly (*p* < 0.05) influenced by NPSB rates, IRS, and their interaction (Table 2).

However, the analysis revealed no statistically significant (*p* > 0.05) three-way interaction (Loc × NPSB × IRS) across all measured traits in this study.

**Figure 2:**
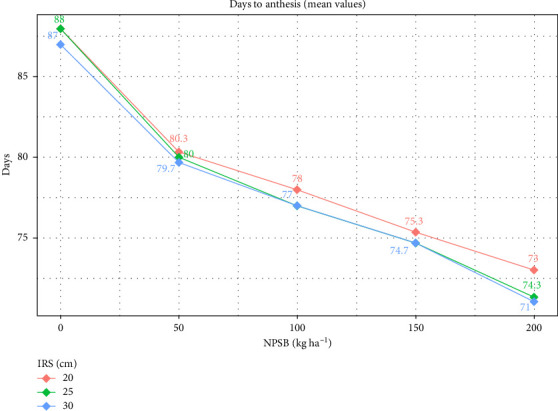
Interaction plot of days to anthesis (AD) by NPSB and intrarow spacing (IRS).

The longest AD (88 days) was observed in the control group (no applied NPSB fertilizer) at 20 and 25 cm IRS. Conversely, the shortest AD (71 days) was recorded with 200 kg·ha^−1^ NPSB at 30 cm IRS, statistically comparable to the 25 cm IRS at the same NPSB dose (Table 3). Adequate nutrient availability and wider IRS reduced interplant competition, leading to increased photosynthate accumulation and accelerated AD. Conversely, lower NPSB rates and narrower IRS (higher population) delayed AD, likely due to reduced photosynthate production during growth and reproductive stages caused by intense interplant competition for resources. Additionally, increased AD at high planting density and low NPSB rates (Figure 2) may indicate stress on maize plants due to intense competition for light, water, and nutrients.

Across locations, DS ranged between 78.83 and 98.83 days. The longest DS occurred with the control group of NPSB at 20 cm interplant spacing (66,666 plants·ha^−1^), statistically similar to 25 cm (53,333 plants·ha^−1^). This aligns with prior research indicating that increased rates of nutritional fertilizer application, predominantly nitrogen, advance tasseling and silking stages [36]. Enhanced nutrient availability, wider IRS, and reduced competition for light and nutrients collectively contributed to earlier anthesis day (AD) and DS silking with higher levels of NPSB. However, Shrestha and Amgain [36] found no significant interaction between nitrogen fertilizer levels and population density on days to anthesis and silking. Conversely, Badu-Apraku et al. [22] demonstrated a significant difference in maize silking days with blended fertilizer application, suggesting that the combination of NPSB fertilizer may promote early crop establishment, rapid growth, and development, thereby reducing silking days.

**Figure 3:**
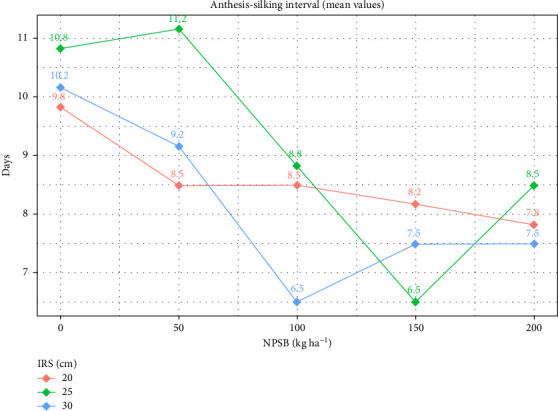
Interaction plot of anthesis-silking interval (ASI) by NPSB and IRS (intrarow spacing).

Across environments, the shortest ASI (6.5 days) was observed with a spacing of 30 cm and the application of 100 kg NPSB·ha^−1^, as well as 25 cm spacing with 150 kg NPSB·ha^−1^ (Table 3). ASI exhibited varied trends with wider spacing and increased NPSB rates. Conversely, the longest ASI (11.17 days) resulted from IRS of 25 cm and 50 kg NPSB·ha^−1^, statistically similar to 20, 25, and 30 cm spacing with no NPSB application (Table 3 and Figure 3). The delay in ASI with higher NPSB application rates indicated vigorous plant growth under such conditions. This aligns with findings by Tokatlidis and Koutroubas [36], who noted that high plant density prolonged the interval for pollen shedding and silk emergence. Similarly, Kebede and Utta [8] reported delayed days to 50% silking with increasing plant density, with the longest silking days observed at the highest plant density of 99,000 plants·ha^−1^.

#### 3.2.2. Growth Parameters

##### 3.2.2.1. PH

**Figure 4:**
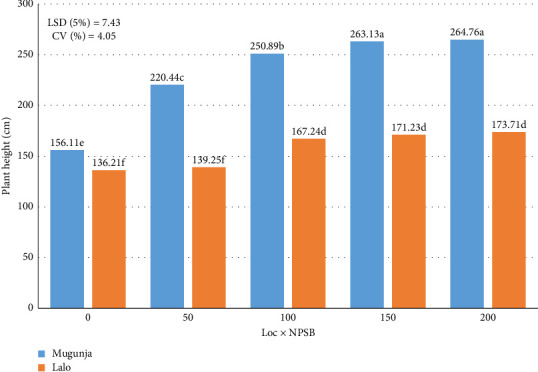
Combined effect of location and NPSB (kg·ha^−1^) on plant height (cm) across locations.

Across locations, the tallest PH at Mugunja (264.76 cm) was achieved with an NPSB rate of 200 kg·ha^−1^, statistically comparable to 150 kg·ha^−1^. Similarly, at Lalo, the tallest PH (173.71 cm) was observed with the highest NPSB rate, not significantly different from 150 to 100 kg·ha^−1^. The shortest PH at Mugunja (156.11 cm) occurred with zero NPSB, while at Lalo, it was with zero NPSB (136.21 cm), statistically similar to 50 kg·ha^−1^ (139.25 cm) (Figure 4). Across locations, increasing NPSB rates from 0 to 200 kg·ha^−1^ corresponded to a rising trend in PH (Figure 2), indicating vigorous plant growth under higher NPSB levels, facilitating enhanced nutrient availability for growth and development. Similar findings were reported by Jena et al. [37], who documented increased PH with higher fertilizer application rates (nitrogen and phosphorus). In another study, Thirupathi et al. [38] found a significant increase in PH with rising sulfur levels up to 60 kg·ha^−1^, with diminishing returns beyond that.

Across various environments, taller plants were observed with decreased IRS (increased plant density). The tallest PHs were recorded at Mugunja (240.77 cm) and Lalo (160.93 cm), both with a 20 cm IRS. Conversely, the shortest PH at Mugunja (222.55 cm) was noted with a wider spacing of 30 cm, while at Lalo, the shortest height occurred with a 30 cm spacing (154.41 cm), statistically comparable to 25 cm (Figure 4). These results support the findings of Belay et al. [7], indicating an interaction effect between location and plant density on PH. Strong competition for light among plants likely contributed to increased height at narrower spacing (higher population). These findings are consistent with conclusions drawn by several authors [23, 24]. Irmak and Djaman [39] similarly reported that IRS influenced maize PH.

##### 3.2.2.2. DPM

**Figure 5:**
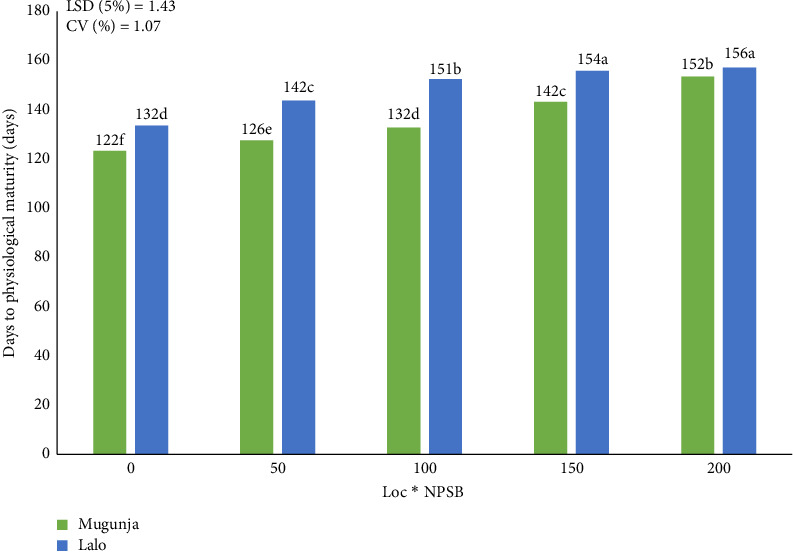
Combined effect of location and NPSB (kg·ha^−1^) on days to physiological maturity.

Across test locations, the mean DPM ranged from 122 to 156 days. In Mugunja, DPM was extended by approximately 19.6% with the application of 200 kg NPSB·ha^−1^ compared to the control (unfertilized plot), while in Lalo, it was delayed by 15% with the same NPSB rate (Figure 5). These findings emphasize the significant influence of higher concentrations of applied NPSB in delaying DPM, a trend consistently observed with increasing NPSB rates at each location. Delayed physiological maturity was consistently observed with higher NPSB rates across various maize hybrids, including Shone-pioneer (30G19) in the Hadero district, as previously reported [18]. Additionally, Orebo et al. [18] documented a delay in DPM with increasing nitrogen doses up to 350 kg·ha^−1^. Regarding location by IRS (Loc x IRS) interactions, at the Lalo site with the highest plant population (20 cm IRS), DPM was slightly longer than at the Mugunja site with the lowest density (30 cm IRS) by a little over 2 weeks (Table 4).

### 3.3. Lodging Characteristics

The interaction between location and IRS significantly (*p* < 0.05) influenced both RL and SL characteristics across environments (Table 5). However, the analysis revealed no statistically significant (*p* > 0.05) two-way (Loc × NPSB) or three-way (Loc × NPSB × IRS) interactions for PH, RL, or SL across all locations in this study.

#### 3.3.1. RL

**Figure 6:**
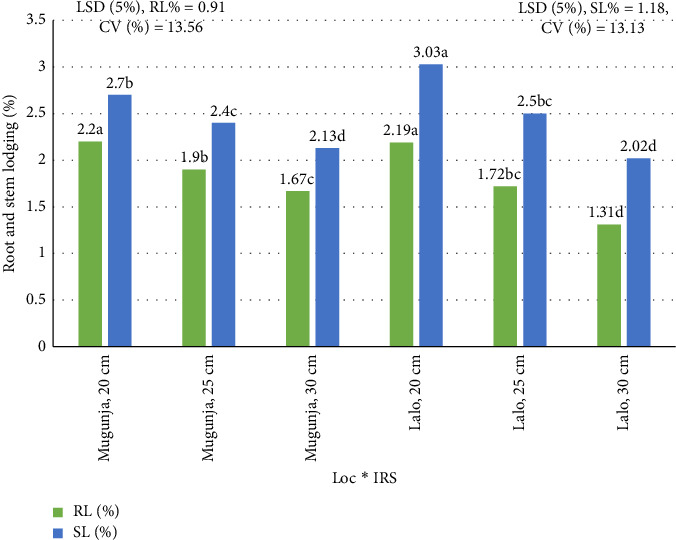
Combined effect of location and intrarow spacing on root lodging (RL) and stem lodging (SL) of maize across locations.

The impact of RL on maize yield and quality is expected to worsen due to changes in agronomic practices such as planting densities and more intense precipitation events, as highlighted by Bernhard and Below [40]. The interaction between location and IRS on RL and SL in maize is illustrated in Figure 6. Mean RL varied from 1.31% to 2.2%. The highest RL was observed at both locations with the highest plant population (20 cm IRS), while the lowest RL was reported with the lowest plant population per hectare (30 cm IRS) (Figure 6). High planting densities increase lodging, disrupt the typical canopy structure, reduce grain production, and diminish photosynthetic activity in the leaves. Consistent with this, Belay and Adare [7] also highlighted a significant relationship between the environment and plant density for maize. Increased planting densities foster intraspecific competitions, impacting various aspects of shoot and root growth [40]. Higher planting densities can diminish the root system's ability to anchor itself effectively due to heightened plant competition. Additionally, according to Liu et al. [40], root lodging tends to increase with rising planting density in maize. Adjusting plant density at the field level is recognized as one of the most efficient strategies for enhancing maize yield per unit area [41].

#### 3.3.2. SL

According to Shi et al. [42], a significant hindrance to enhancing grain output in modern maize production is SL. Across location, SL averaged between 2.02% and 3.03% (Figure 4). The highest SL was observed at the Mugunja site with the highest population (20 cm IRS), while the lowest SL was found at the Lalo site with the smallest population (30 cm IRS) (Figure 6). Generally, SL decreases as the number of plants·ha^−1^ (at wider IRS) decreases at each site. This trend in SL, concerning plant population, aligns with the findings of Shah et al. [43], who reported that SL is more prevalent in areas with dense plant populations. High plant density can render plants more susceptible to lodging due to elongated stems, reduced diameter, and thinner walls, which may compromise flexural rigidity and resistance to breakage [43]. Moreover, increased plant density results in taller plants with smaller stem diameters and longer internodes, thereby increasing the likelihood of SL [44]. The heightened competition for resources among individual plants due to increased plant density also contributes to maize stems becoming thinner and more prone to lodging [44].

### 3.4. Ear Characteristics

#### 3.4.1. Number of EPP

**Figure 7:**
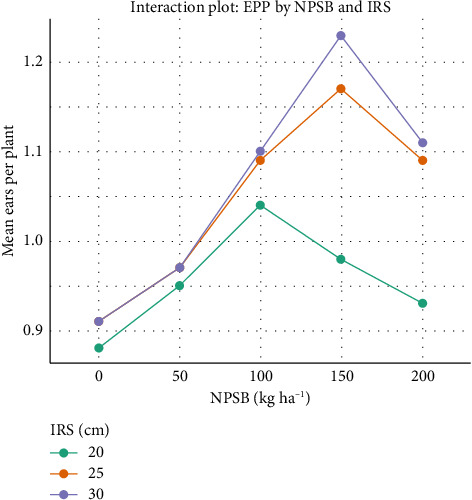
Interaction plot of ears per plant (EPP) by NPSB and IRS (intrarow spacing).

The interaction between NPSB and IRS significantly (*p* < 0.01) influenced the number of EPP across locations (Table 6). However, the interaction of Loc × IRS was not significant for EPP, EL, and EW. The highest EPP (1.23) was observed with 150 kg NPSB·ha^−1^ and 25 cm IRS, but it did not significantly differ from EPP at the same NPSB rate with 30 cm IRS. The lowest EPP was recorded with zero NPSB·ha^−1^ at 20 cm IRS, and there was no notable difference observed with the same NPSB rate at 25 and 30 cm IRS, nor with 200 kg NPSB·ha^−1^ at 20 cm IRS. A 28.46% increase in EPP was noted with 150 kg NPSB·ha^−1^ at 30 cm spacing compared to the lowest EPP at 20 cm IRS with zero NPSB·ha^−1^. Overall, EPP generally increased with higher IRS and NPSB levels, but at 150 kg NPSB·ha^−1^, EPP declined despite increases in population and NPSB rate (Table 7 and Figure 7). These findings align with Golla et al. [45], who observed that increased plant spacing and higher NPSB rates led to a significant increase in EPP.

#### 3.4.2. EL

**Figure 8:**
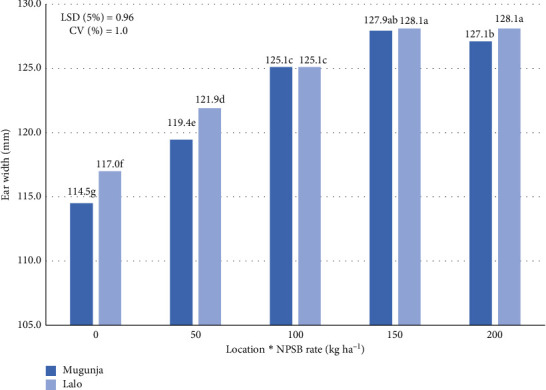
Effect of location by NPSB interaction on ear length (EL) of maize across locations.

Across environments, the Loc × NPSB interaction significantly influenced EL (Table 6). EL ranged from 114.5 to 128.11 mm across environments, with a mean of 123.43 mm (Figure 5). The Lalo site achieved the maximum EL (128.11 mm) with both 150 and 200 kg NPSB·ha^−1^, while the lowest EL was observed with the control NPSB rate. Similarly, at Mugunja, the highest EL (127.94 mm) was attained with 150 kg NPSB·ha^−1^, with no significant difference at 200 kg NPSB·ha^−1^, and the lowest was observed with the control treatment (no NPSB application). However, at both locations, applying 200 kg NPSB·ha^−1^ did not result in appreciable differences compared to 150 kg·ha^−1^ (Figure 8). Ear length increased with increasing NPSB rate from 0 to 150 kg NPSB ha-1 at both sites but did not significantly increase at 200 kg NPSB·ha^−1^ (Figure 8). Enhanced nutrient availability in the soil system likely allowed the maize plant to achieve its maximum yield potential and grow longer ears under higher NPSB rates, explaining the increase in EL with higher NPSB rates at both locations. These findings confirm previous research on N fertilizer use by Golla et al. [45] and blended NPSB fertilizer by Orebo et al. [18] regarding the same trait.

#### 3.4.3. EW

**Figure 9:**
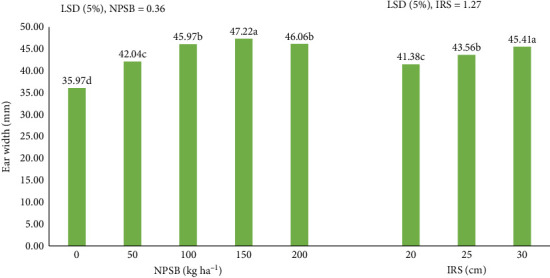
Effect of NPSB rates IRS on ear width (EW) of maize hybrid across environments.

Across environments, the main effects of NPSB and IRS significantly (*p* < 0.01) influenced EW (Table 6) and are depicted in Figure 6. A linear increase in EW was observed with higher NPSB rates. The application of 150 kg NPSB·ha^−1^ resulted in the widest EW (47.22 mm), while the control treatment (without applied NPSB) had the narrowest EW (35.97 mm). However, no significant difference in EW was found between applications of 100 and 200 kg NPSB·ha^−1^ (Figure 9). The rise in EW in response to NPSB rates may be attributed to enhanced nutrient availability in the soil system, enabling the maize plant to reach its maximum yield potential and produce wider ears under higher rate conditions. These findings align with Tadesse and Sultan [46]. Additionally, the widest ears (45.41 mm) were measured at the widest IRS (30 cm), while the narrowest ears (41.38 mm) were measured at the narrowest IRS (20 cm) (Figure 9).

### 3.5. AGBM, HI, and GY

#### 3.5.1. AGBM

The interaction of NPSB × IRS and Loc × NPSB effects significantly (*p* < 0.05) affected AGBM across locations (Table 8). However, the interaction of Loc × IRS was not significant (*p* > 0.05) for AGBM, HI, and GY combined across environments. The largest AGBM (25.68 t·ha^−1^) was found in the Lalo site and the Mugunja site, both at 150 kg NPSB·ha^−1^, which is comparable to 200 kg NPSB·ha^−1^ at the same location (Table 9). While the maximum AGBM at the Lalo location was 46% higher than the zero NPSB rate, the maximum AGBM at the Mugunja location was 53% higher than the lowest AGBM at the control NPSB level. These results demonstrate that the NPSB rate considerably impacted the AGBM yield of maize at both sites. In agreement, previous studies by Belay and Adare [7]; Tadesse and Sultan [46] and Kebede and Utta [8] analogously reported an increase in maize AGBM output as the NPS rate increased from 50 to 150 kg·ha^−1^. According to Mengistu [2] a rising trend in AGBM yield at rising NPSB rates, the maximum at 200 kg NPSB·ha^−1^, provides additional support for the study's findings.

**Figure 10:**
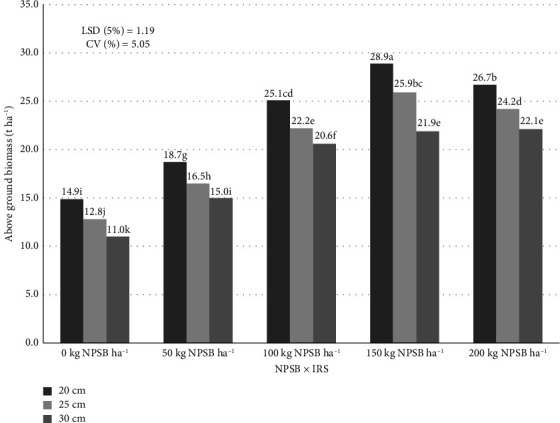
Combined effect of NPSB and IRS on above ground biomass (AGBM) yield of maize.

The highest AGBM (28.92 t·ha^−1^) was achieved with 150 kg NPSB·ha^−1^ and a 20 cm IRS, followed by 26.67 t·ha^−1^ with 200 kg NPSB·ha^−1^ at the same spacing, while the lowest (11.02 t·ha^−1^) occurred with 30 cm spacing and no NPSB (Figure 10). These results indicate that higher planting densities and adequate nutrient supply enhance biomass production, consistent with findings by Mahdi and Ismail [47], Ren et al. [48], and Meng et al. [49], who reported improved nitrogen use efficiency and biomass accumulation under similar conditions. The AGBM in this study, however, dropped at 200 kg NPSB·ha^−1^ with wider IRS (30 cm) and increased up to 150 kg NPSB·ha^−1^ in conjunction with 20 and 25 cm IRS (Figure 10). Such a result suggested that adding more NPSB fertilizer above 150 kg·ha^−1^ did not result in significant improvement in AGBM. According to Belay et al. [7], AGBM in maize increased when nitrogen fertilizer rate increased from 43.5 to 87 kg N at each NPS level before decreasing afterwards.

#### 3.5.2. HI

**Figure 11:**
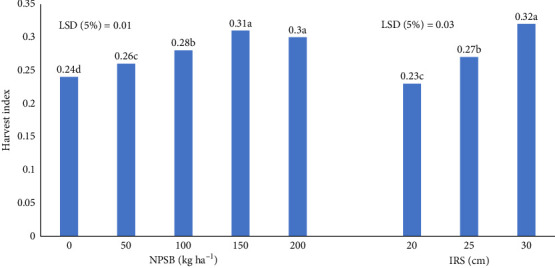
Effect of NPSB rates and IRS on harvest index (HI) of maize.

The HI indicates a plant's efficiency in converting dry matter into marketable produce. Significant (*p* ≤ 0.05) variations in HI were found due to the main effects of NPSB and IRS (Table 8). The highest HI (31%) occurred at 150 kg NPSB·ha^−1^, equivalent to 200 kg·ha^−1^ (Figure 11), suggesting enhanced nutrient availability from NPSB, leading to better crop growth. Conversely, the control NPSB rate had the lowest HI (24%). The widest IRS (30 cm) resulted in the highest HI (32%), while the narrowest (20 cm) had the lowest (23%) (Figure 11). HI increased linearly with wider IRS. These findings align with previous studies by Dhaliwal and Williams [41]; Zhai et al. [50] and Tadesse and Sultan [46], who reported that increased nutrient application boosts HI up to a certain threshold, beyond which it declines.

#### 3.5.3. GY

**Figure 12:**
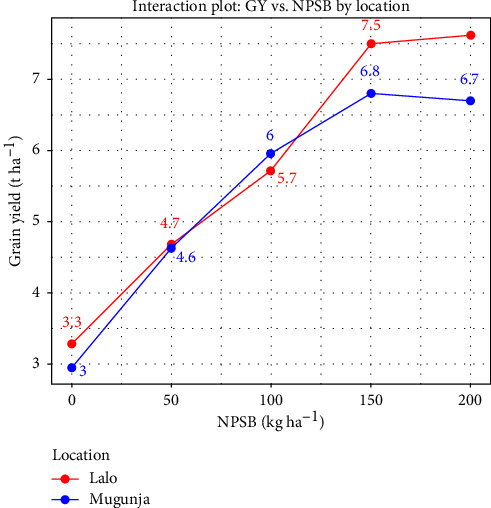
Interaction plot of grain yield (GY) vs NPSB by location.

GY was significantly influenced by the interaction between location and NPSB application (*p* < 0.01) (Table 8), indicating a dependency of applied NPSB rate on prevailing environmental conditions. Across locations, GY ranged from 2.95 to 7.63 t·ha^−1^. In Mugunja, the highest GY was achieved with a NPSB rate of 200 kg·ha^−1^, statistically equivalent to the yield obtained with NPSB at 150 kg·ha^−1^. Similarly, at the Lalo site, the highest yield (7.63 t·ha^−1^) was obtained with a NPSB rate of 200 kg·ha^−1^, statistically comparable to the yield achieved with at 150 kg NPSB·ha^−1^. In contrast, at both sites, the lowest yield was obtained from the unfertilized plots (Table 9). Mean GYs were 56.6% higher at Mugunja and 57% higher at Lalo compared to unfertilized plots at each respective location. At both sites, GY exhibited linear growth up to 150 kg NPSB·ha^−1^ (Figure 12). Beyond this rate, there was no further significant increase in GY, indicating that NPSB at 150 kg·ha^−1^ is the optimal rate for achieving maximum GY of maize in the study areas. Consistent with our study, Abera et al. [6] and Belay and Adare [7] observed an increase in GY in response to an increase in nitrogen and NPS fertilizer rates up to a certain threshold, beyond which no further change was observed.

### 3.6. Partial Budget Analysis

Maize production costs under varying fertilizer management schemes led to fluctuations in total production expenses across treatments. At the Mugunja site, the treatment utilizing 150 kg·ha^−1^ of NPSB fertilizer resulted in the highest NB of 88,957.52 Birr·ha^−1^, boasting an MRR of 1364.34%. Conversely, control plots with 0 kg·ha^−1^ of NPSB fertilizer exhibited the lowest NB at 40,789.9 Birr·ha^−1^. Similarly, at the Lalo site, applying 200 kg·ha^−1^ of NPSB fertilizer yielded the highest NB of 99,568.28 Birr·ha^−1^, with an MRR of 143%. Moreover, employing 150 kg·ha^−1^ of NPSB fertilizer at the same site produced a substantial NB of 98,318 Birr·ha^−1^, accompanied by an impressive MRR of 2933.16%. In contrast, control plots at both sites consistently yielded the lowest NBs, emphasizing the critical role of tailored fertilizer application strategies based on site-specific conditions.

## 4. Conclusion

This study explored the combined effects of blended NPSB fertilizer and IRS on the performance of an improved maize hybrid (*Shone*) in two distinct production settings. Findings indicated significant impacts on growth, phenology, lodging, yield, and yield components due to the main effects or combinations of NPSB, IRS, location, and their interactions. Notably, phonological traits were mainly influenced by NPSB and IRS interactions, while location affected days to maturity and lodging characteristics in conjunction with plant population. Ear characteristics and AGBM were also influenced by NPSB fertilizer rates, location, and IRS interactions. Across locations, application of NPSB fertilizer at 150 kg·ha^−1^ at 30 cm IRS along with 100 kg·ha^−1^ urea fertilizer supplement resulted in maximum economic benefit of maize hybrid than other treatments and recommended for the study areas than blanket recommendations.

## Figures and Tables

**Table 1:** Selected physicochemical properties of the experimental sites before planting.

Soil properties	Mugunja	Lalo	Status	Remark
Particle size distribution (%)				
Sand	33	57		
Silt	28	29		
Clay	39	14		
Textural class	Clay loam	Sandy loam		
BD (g·cm^−3^)	1.2	1.3		
pH	5.94	5.97	Moderate acidic	[32]
%OC	2.2	2.3	Low	[33]
%TN	0.17	0.19	Low	[33]
C:N	12.94	12.1		
Av.P (Ppm)	10.35	9.74	Low	[24]
Av. Ca (meq/100 g)	10.8	12.1		
Av. Mg (meq/100 g)	1.95	2.1		
Av.K (meq/100 g)	0.84	0.86		
CEC (meq/100 g)	23.94	22.14		
Av.SO_4_ (mg·kg^−1^)	11.16	11.23	Low	[34]
Av. B (ppm)	0.58	0.45	Medium and low	[35]

*Note:* Where %OC = per cent of organic carbon, %TN = per cent of total nitrogen, C:N = carbon to nitrogen ratio, Av. P. Ppm = available phosphorus in parts per million, Av. Ca = available Ca, Av. Mg = available magnesium, Av. K = available K.

Abbreviations: BD, bulk density; CEC, cation exchange capacity; pH, power of hydrogen.

**Table 2:** Effect of blended NPSB fertilizer, intrarow spacing, and location on maize phonological characteristics.

Sources of variation	AD	DS	ASI	DPM
Location (Loc)	^∗∗∗^	^∗∗∗^	^∗∗∗^	^∗∗∗^
NPSB	^∗∗∗^	^∗∗∗^	^∗∗∗^	^∗∗∗^
Intrarow spacing (IRS)	^∗∗∗^	^∗∗∗^	^∗^	^∗^
Loc × IRS	ns	ns	ns	^∗∗^
Loc × NPSB	ns	ns	ns	^∗∗∗^
NPSB × IRS	^∗∗∗^	^∗^	^∗∗∗^	ns

*Note:* AD, days to anthesis.

Abbreviations: ASI, anthesis-silking interval; DPM, days to physiological maturity; DS, days to silking; ns, not significant.

^∗^ *p* ≤ 0.05.

^∗∗^ *p* ≤ 0.01.

^∗∗∗^ *p* ≤ 0.001.

**Table 3:** Effect of NPSB and IRS interaction on days to anthesis, days to silking, and anthesis-silking interval on maize hybrid across two locations.

NPSB (kg·ha^−1^)	IRS (cm)	Traits		
		AD (days)	DS (days)	ASI (days)
0	20	88.00^a^	98.83^a^	9.83^abc^
	25	88.00^a^	97.83^a^	10.83^a^
	30	87.00^b^	97.10^a^	10.17^ab^

50	20	80.33^c^	91.50^b^	8.50^c-e^
	25	80.00^c^	88.83^c^	11.17^a^
	30	79.67^c^	88.50^c^	9.17^b-d^

100	20	78.00^d^	86.50^d^	8.50^c-e^
	25	77.00^e^	85.83^d^	8.83^b-e^
	30	77.00^e^	83.50^e^	6.50^f^

150	20	75.33^f^	82.83^e^	8.17^de^
	25	74.67^f^	82.17^ef^	6.50^f^
	30	74.67^f^	81.83^ef^	7.50^ef^

200	20	73.00^g^	80.83^fg^	7.83^d-f^
	25	71.33^h^	79.50^gh^	8.50^c-e^
	30	71.00^h^	78.83^h^	7.50^ef^

LSD (5%)		0.94	1.8	1.43

CV (%)		1.03	1.78	14.34

*Note:* AD, days to anthesis; means in the same column and the same letters are not significantly different at 5% level of probability; LSD, least significant difference at 5% level of probability and CV (%), coefficient of variation.

Abbreviations: ASI, anthesis-silking interval; DS, days to silking; IRS, intrarow spacing.

**Table 4:** Effect of location and intrarow spacing on plant height and days to physiological maturity across locations.

Location	IRS	PH (cm)	DPM (days)
Mugunja	20	240.77^a^	134.80^d^
	25	229.88^b^	134.67^d^
	30	222.55^c^	134.93^d^

Lalo	20	160.93^d^	145.78^c^
	25	157.25^de^	147.35^b^
	30	154.41^e^	148.47^a^

LSD (5%)		28.76	5.52

*Note:* Means in the same column and the same letters are not significantly different at 5% level of probability. LSD, least significant difference at 5% level of probability.

Abbreviations: DPM, day to physiological maturity; IRS, intrarow spacing; PH, plant height.

**Table 5:** Combined analysis of variance for blended NPSB, intrarow spacing, and location effects and their interaction on plant height, root, and stem lodging at Mugunja and Lalo.

Sources of variation	PH (cm)	RL (%)	SL (%)
Location (Loc)	^∗∗∗^	^∗∗^	ns
NPSB	^∗∗∗^	^∗∗∗^	^∗∗∗^
Intra-row spacing (IRS)	^∗∗∗^	^∗∗∗^	^∗∗∗^
Loc × IRS	^∗^	^∗^	^∗^
Loc × NPSB	^∗∗∗^	ns	ns
CV (%)			

Abbreviations: IRS, intrarow spacing; ns, not significant; PH, plant height; RL, root lodging; SL, stalk lodging.

^∗^ *p* ≤ 0.05.

^∗∗^ *p* ≤ 0.01.

^∗∗∗^ *p* ≤ 0.001.

**Table 6:** Combined analysis of variance for blended NPSB, intrarow spacing, and location effects and their interaction on ears per plant (EPP), ear length (EL), and ear width (EW) of maize across locations.

Sources of variation	EPP	EL	EW
	#	(mm)	(mm)
Location (Loc)	ns	^∗∗∗^	ns
NPSB	^∗∗∗^	^∗∗∗^	^∗∗∗^
Intrarow spacing (IRS)	^∗∗∗^	^∗∗∗^	^∗∗∗^
Loc × NPSB	ns	^∗∗∗^	ns
NPSB × IRS	^∗∗∗^	ns	ns
CV (%)	5.20	0.96	3.56

Abbreviations: EL, ear length; EPP, ears per plant; EW, ear width; ns, not significant.

^∗^ *p* ≤ 0.05.

^∗∗^ *p* ≤ 0.01.

^∗∗∗^ *p* ≤ 0.001.

**Table 7:** Effect of NPSB and intrarow spacing (IRS) interaction on number of ears per plant (EPP) across environments.

NPSB (kg·ha^−1^)	IRS (cm)		
	20	25	30
0	0.88^h^	0.91^gh^	0.91^gh^
50	0.95^fg^	0.97^fg^	0.97^fg^
100	1.04^de^	1.09^cd^	1.10^c^
150	0.98^ef^	1.17^ab^	1.23^a^
200	0.93f^gh^	1.09^cd^	1.11^bc^
LSD (5%)	1.19		
CV	5.20		

*Note:* means with same letters are not significantly different at 5% level of probability. LSD, least significant difference at 5% level of probability.

Abbreviation: IRS, intrarow spacing.

**Table 8:** Mean squares from combined analysis of variance for grain yield, above ground biomass and harvest index.

Sources of variation	GY (t·ha^−1^)	AGBM (t·ha^−1^)	HI
Location (Loc)	^∗∗^	ns	ns
NPSB	^∗∗∗^	^∗∗∗^	^∗∗∗^
Intra-row spacing (IRS)	^∗∗^	^∗∗∗^	^∗∗∗^
Loc × NPSB	^∗^	^∗∗^	ns
NPSB × IRS	ns	^∗^	ns
CV (%)	11.08	5.05	ns

*Note:* ns = not significant at *p* = 0.05. ABGM, above ground biomass.

Abbreviations: CV, coefficient of variation; GY, grain yield; HI, harvest index.

^∗^ *p* ≤ 0.05.

^∗∗^ *p* ≤ 0.01.

^∗∗∗^ *p* ≤ 0.001.

**Table 9:** Effect of location × NPSB interaction on grain yield (GY) and above ground biomass (AGBM) of maize across locations.

Location	NPSB	GY	AGBM
	(kg·ha^−1^)	(t·ha^−1^)	(t·ha^−1^)
Mugunja	0	2.95^e^	12.04^f^
	50	4.63^d^	16.57^d^
	100	5.95^c^	22.63^c^
	150	6.80^b^	25.68^a^
	200	6.70^b^	24.92^a^

Lalo	0	3.28^e^	13.73^e^
	50	4.68^d^	16.96^d^
	100	5.72^c^	22.63^c^
	150	7.50^a^	25.48^a^
	200	7.63^a^	23.74^b^

LSD (5%)		0.58	0.98

*Note:* Means in a column with same letters are not significantly different at *p* = 0.05. AGBM, above ground biomass.

Abbreviation: GY, grain yield.

## Data Availability

The data used to support the findings of this study are available from the corresponding author upon request.
